# Resource utilization and outcomes of intoxicated drivers

**DOI:** 10.1186/1752-2897-4-9

**Published:** 2010-08-05

**Authors:** Robert A Cherry, Pamela A Nichols, Theresa M Snavely, Lindsay J Camera, David T Mauger

**Affiliations:** 1 Penn State Milton S. Hershey Medical Center, Department of Surgery, Shock Trauma Center, Hershey, Pennsylvania 17033, USA; 2 Penn State Milton S. Hershey Medical Center, Public Health Sciences, Hershey, Pennsylvania 17033, USA

## Abstract

**Background:**

The high risk behavior of intoxicated drivers, impaired reaction time, lack of seat belt use, and increased incidence of head injury raises questions of whether pre-hospital use of alcohol leads to a higher injury severity score and worse clinical outcomes. We therefore compared intoxicated and non-intoxicated drivers of motor vehicle crashes with respect to outcome measurements and also describe the resources utilized to achieve those outcomes at our Level 1 trauma center.

**Methods:**

Retrospective descriptive study (Jan 2002-June 2007) of our trauma registry and financial database comparing intoxicated drivers with blood alcohol levels (BAC) > 80 mg/dl (ETOH > 80) with drivers who had a BAC of 0 mg/dl (ETOH = 0). Drivers without a BAC drawn or who had levels ranging from 1 mg/dL to 80 mg/dL were excluded. Data was collected on demographic information (age, gender, injury severity score or ISS), outcome variables (mortality, complications, ICU and hospital LOS, ventilator days) and resource utilization (ED LOS, insurance, charges, costs, payments). Statistical analysis: p < 0.05 vs. ETOH > 80; stratified chi square.

**Results:**

Out of 1732 drivers, the combined study group (n = 987) of 623 ETOH = 0 and 364 ETOH > 80 had a mean age of 38.8 ± 17.9, ISS of 18.0 ± 12.1, and 69.8%% male. There was no difference in ISS (p = 0.67) or complications (p = 0.38). There was a trend towards decreased mortality (p = 0.06). The ETOH = 0 group had more patients with a prolonged ICU LOS (≥ 5 days), ventilator days (≥ 8 days), and hospital LOS (> 14 days) when compared to the ETOH > 80 group (p < 0.05). The ETOH > 80 group tended to be self pay (4.9% vs. 0.7%, p < 0.5) and less likely to generate payment for hospital charges (p < 0.5). Hospital charges and costs were higher in the ETOH = 0 group (p < 0.5).

**Conclusions:**

The data suggests that intoxicated drivers may have better outcomes and a trend towards reduced mortality. They appeared to be less likely to have prolonged hospital LOS, ICU LOS, and ventilator days. We also observed that intoxicated drivers were more likely to be self-pay, less likely to have charges > $50K, and less likely to pay ≥ 90% of the charges. Further research using multivariable analysis is needed to determine if these apparent outcomes differences are driven by acute intoxication, and the tendency for endotracheal intubation and ICU admission, rather than injury severity.

## Background

The prevalence of motor vehicle crashes remains a major public health concern and is a leading cause of morbidity and mortality in the United States. There were 38,588 fatalities and 1,746,000 injuries associated with motor vehicle crashes that were reported by police in 2006 [1]. Many of these motor vehicle crashes are alcohol-related and accounted for 13,470 fatalities [2]. The National Highway Traffic Safety Administration (NHTSA) has reported that 20% of fatal crashes in 2005 involved drunk drivers (BAC of ≥ 80 mg/dL) [3]. According to NHTSA, alcohol-related crashes account for $51.1 billion or 22% of the economic costs. Approximately 75% of these costs involve crashes in which a driver or non-occupant had a BAC of at least 100 mg/dl. The high percentage of intoxicated drivers is therefore a major driver of the socioeconomic costs involving motor vehicle crashes.

Moreover, there have been a number of studies that have found an association between drinking and driving and an increased risk for motor vehicle injury [4-6]. The Insurance Institute for Highway Safety reported that a BAC as low as 20 mg/dL increases the likelihood of a crash while operating a motor vehicle [5]. The probability of a crash increases significantly at 50 mg/dL and rises rapidly at levels greater than 100 mg/dL. Even among people who are 55 years and older, drinking history (12 or more drinks prior to death) is associated with fatality from a motor vehicle crash [7].

The incidence of alcohol-impaired driving, and the risk of injury from alcohol-related motor vehicle crashes, is also strongly associated with binge drinking [8,9]. Other predictors for a recurrent motor vehicle crash included age < 32 years old, male sex, nighttime crash, and a BAC > 50 mg/dl [6]. Drivers who tested positive for blood alcohol are noncompliant with seat belt use [10-13] and are more likely to have suffered a head injury [11,13,14].

The high risk behavior of intoxicated drivers, impaired reaction time, lack of seat belt use, and increased incidence of head injury all raises the question of whether pre-hospital use of alcohol leads to a higher injury severity score, and therefore worse clinical outcomes. We therefore compared intoxicated and non-intoxicated drivers of motor vehicle crashes with respect to outcome measurements and also describe the resources utilized to achieve those outcomes at our Level 1 trauma center.

## Methods

This was a retrospective descriptive study conducted at our Level 1 trauma center from January 2002 through June 2007. Adult trauma patients who were 18 years of age and older, operated a motor vehicle, and involved in a crash were identified using our trauma registry (Collector, Digital Innovation, Forest Hill, Maryland). Drivers were identified and differentiated from passengers based on EMS and/or emergency department records. Patients with documented blood alcohol tests were identified by accessing the electronic medical record (Cerner Corporation, Kansas City, Missouri). There is mandatory reporting of all suspected or confirmed cases of alcohol intoxication to the Pennsylvania Department of Transportation. At our institution, blood alcohol concentrations are obtained on suspected cases of alcohol intoxication if the laboratory finding might affect therapeutic decision-making or result in a referral to a drug and alcohol counselor.

We compared intoxicated drivers with alcohol concentrations > 80 mg/dl (ETOH > 80) with drivers who had alcohol concentrations of 0 mg/dl (ETOH = 0). Drivers without an ETOH level drawn or who had results ranging from 1 mg/dl to 80 mg/dl were excluded from the study. The legal limit for blood alcohol concentrations in Pennsylvania is 80 mg/dL. Patients without a blood alcohol concentration drawn and inter-facility transfers were excluded.

Data was collected on outcome measurements such as mortality, complications, ICU and hospital length of stay or LOS, and ventilator days. Resource utilization was assessed by obtaining variables on time spent in the emergency department (ED), insurance type, inpatient charges, medical costs, and payments made (PMT). Information on discharge destination from the ED and the hospital was also collected. The Cochran-Mantel-Haenszel Chi-square test, stratified by age, gender, and injury severity score (ISS), was used to compare the ETOH > 80 group against the ETOH = 0 group with respect to these outcomes.

All analyses were carried out using SAS Version 9 (SAS Institute Inc., Cary, NC). All p values < 0.05 were used to denote significant differences between groups. The Cochran-Mantel-Haenszel Chi-square test, stratified by age, gender, and injury severity score (ISS), was used to compare the ETOH > 80 group against the ETOH = 0 group with respect to these outcomes and odds ratios were calculated for binary outcomes. Both stratified and unstratified odds ratios with 95% confidence intervals were constructed. The study protocol was approved by the Institutional Review Board at the Penn State Milton S. Hershey Medical Center.

## Results

There were 1,732 adult drivers of motor vehicles identified during the study period who were evaluated at our trauma center. The combined study group consisted of 623 patients with a BAC of 0 mg/dL (ETOH = 0 group) and 364 patients with a BAC > 80 mg/dL (ETOH > 80 group). Collectively, these patients had a mean age of 38.8 ± 17.9 years, an ISS of 18.0 ± 12.1, and were 69.8% male. There was no significant difference is ISS among the two groups (p < 0.66), but the ETOH > 80 group was younger and had more men (p < 0.05).

The ETOH = 0 group tended to have a prolonged ED LOS (≥ 220 minutes, see Table 1). The ETOH > 80 group was more likely to be admitted to the ICU (41.3% vs. 33.7%, p < 0.05) and intermediate care unit (18.2% vs. 16.1%, p < 0.05) from the ED, and the ETOH = 0 group was more likely to go to the operating room (Table 2). Despite fewer admissions to the ICU, the ETOH = 0 group had significantly prolonged ICU LOS (≥ 5 days), ventilator days (≥ 8 days), and hospital LOS (> 14 days) when compared to the ETOH > 80 group (p < 0.05) (Table 2). There was no significant difference in the number of deaths for the ETOH > 80 group, but there was a trend towards decreased mortality (p = 0.06).

**Table 1:** Outcome measurements of intoxicated vs. non-intoxicated drivers

	**ED ≥ 220 min**.	ICU LOS ≥ 5days	Vent days ≥ 8	Hospital LOS > 14 days
ETOH = 0	25.6%*, 159/598	19.0%*, 118/621	10.5%*, 65/618	17.8%*, 111/623
ETOH > 80	22.5%, 78/346	13.5%, 49/364	5.0%, 18/361	11.5%, 42/364

* - p < 0.05 vs. ETOH > 80

ED - emergency department

ICU - intensive care unit

Vent - ventilator

LOS-length of stay

ETOH = 0-blood alcohol level of 0 mg/dL

ETOH > 80

blood alcohol level > 80 mg/dL

**Table 2:** Post emergency department destination

	ICU	Medical/Surgical Unit	Intermediate Care Unit	OR	Morgue
ETOH = 0	33.7%, n = 209	28.0%, n = 174	16.1%, n = 100	22.1%, n = 137	0.2%, n = 1
ETOH > 80	41.3%, n = 150	21.8%, n = 79	18.2%, n = 66	17.9%, n = 65	0.8%, n = 3
Total	359	253	166	202	4

ICU - intensive care unit

OR - operating room

LOS- length of stay

ETOH = 0 - blood alcohol level of 0 mg/dL

ETOH > 80

blood alcohol level > 80 mg/dL

The ETOH > 80 group tended to be self pay (4.9% vs. 0.7%, p < 0.5) and less likely to generate payment for hospital charges (p < 0.5) (Table 3). Of note, hospital charges and medical costs were significantly higher in the ETOH = 0 group (p < 0.5). The ETOH > 80 group was significantly more likely to go home (76.5% vs. 65.6%) and less likely to be discharged to a skilled nursing facility (1.5% vs. 5.2%) and a rehabilitation center (15.7% vs. 25.6%) (Table 4).

**Table 3:** Financial variables of intoxicated vs. non-intoxicated drivers

	Self-pay	Charges > $50K	Costs > $25K	PMT/Charge > 0.9
ETOH = 0	0.7%*, 3/416	29.6%*, 123/416	25.5%, 106/416	28.4%*, 118/416
ETOH > 80	4.9%, 10/203	20.8%, 42/202	18.1%, 38/202	16.3%, 33/202

* - p < 0.05 vs. ETOH > 80

PMT - payment

ETOH = 0 - blood alcohol level of 0 mg/dL

ETOH > 80

blood alcohol level > 80 mg/dL

**Table 4:** Most common discharge destinations

	Home	Rehabilitation Center	Skilled Nursing Facility	Legal Authority	AMA	Hospital Transfer	Psychiatric Facility
ETOH = 0	65.6%, n = 395	25.6%, n = 154	5.2%, n = 31	0.2%, n = 1	0.5%, n = 3	1.0%, n = 6	0.2%, n = 1
ETOH > 80	76.5%, n = 264	15.7%, n = 54	1.5%, n = 5	2.0%, n = 7	1.5%, n = 5	0.3%, n = 1	1.5%, n = 5
Total	659	208	36	8	8	7	6

AMA - against medical advice

ETOH = 0 - blood alcohol level of 0 mg/dL

ETOH > 80

blood alcohol level > 80 mg/dL

## Discussion

Our study describes several unexpected observations among intoxicated drivers with respect to resource utilization and outcome measurements. These results underscore several areas requiring further discussion, including the burden that alcohol misuse and abuse may place on hospital resource utilization, and whether alcohol intoxication is a potential predictor of clinical outcome. In addition, the financial implications that intoxicated drivers have on our Level 1 trauma center becomes more of an issue after reviewing the data in this study.

Although our study did not specifically control for ISS, the data did not show differences in ISS between BAC > 80 and BAC = 0 groups. The groups were also comparable in that there were no differences in complications or mortality. Nevertheless, there have been conflicting reports in the literature with regards to injury severity for occupants involved in alcohol-related motors vehicles crashes. Ward and his colleagues, for instance, studied 1,198 trauma patients with evidence of alcohol use and found no difference in the severity of injury compared with those with a negative blood alcohol concentrations [15]. In another study, Smink and associates also found no difference in the mean ISS scores between drivers who test negative for alcohol, and those who do not [16]. On the other hand, Brown et. al. found that ISS was significantly higher for occupants of vehicles that were operated by intoxicated drivers with a blood alcohol concentrations of 100 mg/dl [17]. Honkanen and his associates also found a positive correlation between injury severity and intoxication among injured occupants of motor vehicles [18].

The issue that emerges is whether there are outcome differences between intoxicated drivers regardless of ISS. Shih and his colleagues investigated 923 injured drivers involved in motor vehicle and motorcycle crashes in which 421 of them had BAC ≥ 50 [19]. Drivers with a BAC ≥ 50 had a significantly higher ISS. However, after a logistic regression analysis, a BAC ≥ 50 was not associated with severe injury as defined by an ISS ≥ 9 or mortality. However, alcohol intoxication (BAC ≥ 50) was a predictor of morbidity. In the Washington State study by Mueller, BAC > 50 mg/dL was also found to be a predictor for morbidity [20]. Age greater than 54 years old and BAC > 50 mg/dL did not predict mortality.

Perhaps one of the more provocative studies was performed by Koval and associates. They performed a recent retrospective study of 67,021 patients in which 38.3% were drivers involved in a MVC [11]. The strongest predictor for mortality was ISS. Other risk factors cited were male sex and age. Most notably, "protective factors" included the presence of alcohol and the use of safety devices. Other published reports also suggest that alcohol intoxication may be protective. For example, outcomes after MVC and isolated severe traumatic brain injury are significantly different depending upon the level of ETOH and not simply upon the presence or absence of serum ETOH [21,22].

One should not necessarily infer from the data described in our study, however, that intoxication is protective for drivers involved in a motor vehicle crash. This study is limited in that logistic regression analysis was not performed. The culling of additional numerical or categorical predictor variables to determine the probability of occurrence is certainly desirable. We believe that a multivariable analysis to determine outcomes differences would best be performed as a prospective study because of the limitations inherent in a retrospective, registry-based investigation. The prospective collection of variables such as vehicle speed and type, daytime vs. nighttime vehicle operation, seat belt use, airbag deployment, driver experience, and distractions (ex. number of occupants) would also be valuable. For these reasons, we have categorized this report as a retrospective descriptive study. Apparent associations due to differences between groups must therefore be interpreted with caution.

Responsibility demands a critical analysis of these observations looking for alterative explanations that require further investigation. For instance, there were an increased number of admissions to the ICU and IMC among the ETOH > 80 group despite no differences in ISS. This contradiction could be explained if a disproportionate number of intoxicated patients required endotracheal tube intubation and a short ICU length of stay due to binge drinking. Patient care would therefore be driven by medical necessity and not by injury severity. The shorter ICU and hospital LOS found in the ETOH > 80 is consistent with this line of reasoning. This argument would suggest that some ICU and IMC admissions among patients with an ETOH > 80 may have been unnecessary. On the other hand, one would have expected to find differences in injury severity score among the two groups if this were the case. Of note, patients with an ETOH = 0 were more likely to go to the operating room and may have been more seriously injured than what was captured by ISS. This might explain why the ETOH > 80 group had fewer admissions to the ICU, and a reduced incidence of prolonged mechanical ventilation.

In our data, ED LOS was also notably shorter in the ETOH > 80 group. The overall relationship between LOS and alcohol intoxication is conflicting in the literature. Brotman and associates found that hospital length of stay among trauma patients who tested positive for alcohol was similar to those who tested negative [23]. In contrast, Mueller found that drivers under the influence of alcohol had longer hospital stays after adjusting for age, gender, and injury severity [20].

We also observed that the ETOH > 80 group was more likely to be self-pay, more likely to be admitted to the ICU and IMC, and less likely to pay for the charges associated with their medical care. Our trauma center is therefore less likely to recover operating costs when caring for intoxicated drivers. If these findings are consistent at other institutions, then the additional services rendered may represent another financial burden for trauma centers, especially those that are already financially troubled. Of note, Mueller and colleagues has already shown that hospital charges in Washington State were greater for drinking drivers when compared to nondrinking divers [20].

There are several other limitations of this study. It is a retrospective study and contains some missing data elements that are inherent to trauma registries in general. There is also a possibility that patients with a BAC = 0 are less likely to be admitted to the hospital than intoxicated patients. This would result in a relatively greater resource utilization among patients with an ETOH > 80. In addition, patients who are discharged directly from the ED are not considered state qualifiers for inclusion in the registry. This may have an impact on ISS between groups, especially if BAC = 0 patients are disproportionately discharged. In addition, because only those patients that were considered registry qualifiers in our state were included, we do not know the actual number of patients that may have been treated and released, with or without a trauma evaluation. Therefore, we do not know the actual ICU days per patient for either group. Furthermore, alcohol screening was not mandatory for all drivers and might introduce a selection bias into the methodology. Finally, since this is a single center study, our findings may not necessarily reflect those seen at other trauma centers.

Further research is needed in this area. For instance, population-based studies that investigate the outcomes of all drivers in a region, including those dead at the scene, would be valuable. Investigations may also be conducted that carefully control for injury mechanism, such as speed, impact location, type of vehicle, and seat belt/air bag protection. Future studies should also consider selecting a primary outcome parameter of interest. For example, propensity scores could be developed and used to predict the need for intubation and prolonged mechanical ventilation based on age, gender, ISS, abbreviated injury score for the head, Glasgow Coma Score, admission blood pressure and arterial pO_2 _(or oxygen saturation), and the presence or absence of rib fractures. Intoxicated patients could then be case-matched with unintoxicated patients having similar propensity scores and assessed for the incidence of intubation and prolonged mechanical ventilation. The sample size needed for such a study is probably too small to be conducted at any single institution.

## Conclusions

Drivers of motor vehicle crashes with a BAC > 80 mg/dL who presented at our trauma center were observed to have better outcomes compared with drivers having no evidence of alcohol use. Intoxicated drivers were described as having lower ICU admission rates, hospital and ICU length of stay, ventilator days, and inpatient charges. Those drivers were also more likely to be discharge to home. The appearance of improved outcomes in intoxicated drivers may have been driven by acute intoxication, and the tendency for endotracheal intubation and ICU admission, rather than injury severity. Further studies are needed to corroborate these observations at other trauma centers.

## Competing interests

The authors declare that they have no competing interests.

## Authors' information

RAC: Study concept and design. RAC, PAN, TMS: Acquisition of data. RAC, DTM, LJC: Analysis and interpretation of data. RAC: Drafting of the manuscript. RAC, PAN, TMS, LJC: Critical revision of the manuscript for important intellectual content. DTM, LJC: Statistical analysis. RAC, PAN, TMS: Administrative, technical, and material support. All authors have read and approved the final manuscript.

## Additional note

The authors are pleased that an invited editorial by Dr. Uli Schmucker [24] has been included with this article. Many of his remarks have been considered during the course of our clinical investigation. We agree with his assessment that further research is needed to determine if acute alcohol intoxication has an impact on trauma outcomes.

## References

[B1] U. S. Department of Transportation Traffic safety facts 2006. A compilation of motor vehicle crash data from the fatality analysis reporting system and the general estimate system 2006 http://www-nrd.nhtsa.dot.gov/Pubs/TSF2006FE.PDF Accessed August 22, 2009

[B2] U.S. Department of Transportation Traffic safety facts 2008 data 2008 http://www-nrd.nhtsa.dot.gov/Pubs/811162.PDF Accessed August 10, 2009

[B3] U.S. Department of Transportation Traffic safety facts 2005. A compilation of motor vehicle crash data from the fatality analysis reporting system and the general estimate system 2005 http://www-nrd.nhtsa.dot.gov/Pubs/TSF2005.PDF Accessed August 2, 2009

[B4] Bell NS Amoroso PJ Yore MM Smith GS Jones BH Self-reported risk-taking behaviors and hospitalization for motor vehicle injury among active duty army personnel Am J Prev Med 2000 18 85 95 10.1016/S0749-3797(99)00168-3 10736544

[B5] Insurance Institute for Highway Safety (IIHS) Fatality facts Arlington, VA 1993

[B6] Fabbri A Marchesini G Dente M Iervese T Spada M Vandelli A A positive blood alcohol concentration is the main predictor of recurrent motor vehicle crash Ann Emerg Med 2005 46 161 167 10.1016/j.annemergmed.2005.04.002 16046947

[B7] Sorock GS Chen LH Gonzalgo SR Baker SP Alcohol-drinking history and fatal injury in older adults Alcohol 2006 40 193 199 10.1016/j.alcohol.2007.01.002 17418699

[B8] Quinlan KP Brewer RD Siegel P Alcohol-impaired driving among U.S. adults, 1993-2002 Am J Prev Med 2005 28 346 350 10.1016/j.amepre.2005.01.006 15831339

[B9] Vingilis E Wilk P Predictors of motor vehicle collision injuries among a nationally representative sample of Canadians Traffic Inj Prev 2007 8 411 418 10.1080/15389580701626202 17994496

[B10] Allen S Zhu S Sauter C Layde P Hargarten S A comprehensive statewide analysis of seatbelt non-use with injury and hospital admissions: new data, old problem Acad Emerg Med 2006 13 427 434 10.1111/j.1553-2712.2006.tb00321.x 16531597

[B11] Koval KJ Cooley M Cantu RV Spratt KF The effects of alcohol on in-hospital mortality in drivers admitted after motor vehicle accidents Bull NYU Hosp Jt Dis 2008 66 27 34 18333825

[B12] Ball CG Kirkpatrick AW Brenneman FD Noncompliance with seat-belt use in patients involved in motor vehicle collisions Can J Surg 2005 48 367 372 16248134 PMC3211890

[B13] Honkanen R Smith G Impact of acute alcohol intoxication on patterns of non-fatal trauma: cause-specific analysis of head injury effect Injury 1991 22 225 229 10.1016/0020-1383(91)90047-I 2071207

[B14] Chen SC Lin FY Chang KJ Body region prevalence of injury in alcohol-and non-alcohol-related traffic injuries J Trauma 1999 47 881 884 10.1097/00005373-199911000-00011 10568716

[B15] Ward RE Flynn TC Miller PW Blaisdell WF Effects of ethanol ingestion on the severity and outcome of trauma Am J Surg 1982 144 153 157 10.1016/0002-9610(82)90617-1 7091524

[B16] Smink BE Movig KL Lusthof KJ De Gier JJ Uges DR Egberts AC The relation between the use of psychoactive substances and the severity of the injury in a group of crash-involved drivers admitted to a regional trauma center Traffic Inj Prev 2008 9 105 108 10.1080/15389580701824443 18398772

[B17] Brown CK Cline DM Factors affecting injury severity to rear-seated occupants in rural motor vehicle crashes Am J Emerg Med 2001 19 93 98 10.1053/ajem.2001.19982 11239249

[B18] Honkanen R Smith GS Impact of acute alcohol intoxication on the severity of injury: a cause-specific analysis of non-fatal trauma Injury 1990 21 353 357 10.1016/0020-1383(90)90117-D 2276795

[B19] Shih HC Hu SC Yang CC Ko TJ Wu JK Lee CH Alcohol intoxication increases morbidity in drivers involved in motor vehicle accidents Am J Emerg Med 2003 21 91 94 10.1053/ajem.2003.50025 12671806

[B20] Mueller BA Kenaston T Grossman D Salzberg P Hospital charges to injured drinking drivers in Washington State: 1989-1993 Accid Anal Prev 1998 30 597 605 10.1016/S0001-4575(98)00017-7 9678213

[B21] Plurad D Demetriades D Cruzinski G Motor vehicle crashes: the association of alcohol consumption with the type and severity of injuries and outcomes J Emer Med 2010 38 12 17 10.1016/j.jemermed.2007.09.048 18547772

[B22] Talving P Plurad D Barmparas G Isolated severe traumatic brain injuries: Association of blood alcohol levels with the severity of injuries and outcomes J Trauma 2010 68 357 362 10.1097/TA.0b013e3181bb80bf 20154549

[B23] Brotman S Indeck MC Leonard D Huber J The study of the relationship between lifestyle characteristic self-reported drinking patterns and trauma Am Surg 1995 61 975 979 7486430

[B24] Schmucker U Editorial Commentary: Resource utilization and outcomes of intoxicated drivers: does evidence of alcohol-impaired driving affect road traffic crash injury outcomes? Journal of Trauma Management and Outcomes 2010 4 2 20687913 10.1186/1752-2897-4-10 PMC2924843

